# *In Silico* Determination of Gas Permeabilities by Non-Equilibrium Molecular Dynamics: CO_2_ and He through PIM-1

**DOI:** 10.3390/membranes5010099

**Published:** 2015-03-10

**Authors:** Hendrik Frentrup, Kyle E. Hart, Coray M. Colina, Erich A. Müller

**Affiliations:** 1 Department of Chemical Engineering, Imperial College London, South Kensington Campus, London SW7 2AZ, UK; E-Mail: hendrik.frentrup10@imperial.ac.uk; 2 Department of Materials Science and Engineering, The Pennsylvania State University, University Park, PA 16802, USA; E-Mails: keh243@psu.edu (K.E.H.); colina@matse.psu.edu (C.M.C.)

**Keywords:** gas permeation, glassy polymer, solution, diffusion, molecular dynamics, non-equilibrium, simulation

## Abstract

We study the permeation dynamics of helium and carbon dioxide through an atomistically detailed model of a polymer of intrinsic microporosity, PIM-1, via non-equilibrium molecular dynamics (NEMD) simulations. This work presents the first explicit molecular modeling of gas permeation through a high free-volume polymer sample, and it demonstrates how permeability and solubility can be obtained coherently from a single simulation. Solubilities in particular can be obtained to a very high degree of confidence and within experimental inaccuracies. Furthermore, the simulations make it possible to obtain very specific information on the diffusion dynamics of penetrant molecules and yield detailed maps of gas occupancy, which are akin to a digital tomographic scan of the polymer network. In addition to determining permeability and solubility directly from NEMD simulations, the results shed light on the permeation mechanism of the penetrant gases, suggesting that the relative openness of the microporous topology promotes the anomalous diffusion of penetrant gases, which entails a deviation from the pore hopping mechanism usually observed in gas diffusion in polymers.

## 1. Introduction

The quest for efficiency improvements of gas separation membrane materials has spawned interdisciplinary research efforts targeted at searching for novel materials that deliver improved performance with a lower economic and environmental footprint [1,2,3]. Pressure-driven gas separations, through versatile and easily-manufactured polymeric membranes with high permeation and selectivity, are of particular interest and highly sought-after for fulfilling those purposes [4,5].

Separating gases through a selective polymer membrane requires the gases to exhibit different rates of permeation through the polymer material. In order to find effective strategies to identify the individual parameters affecting permeability, it is important to reach a detailed understanding of permeation mechanisms in porous polymers. Differences in permeability stem from variations in the sorption and diffusion of gases in the polymer, which, in turn, are brought about by variations in polymer chemistry, microstructure or pore topology.

Molecular simulations have been instructive in investigating transport properties in structured molecular materials, such as zeolites [6], metal-organic frameworks [7] and carbon structures [8]. Molecular dynamics (MD) is the most proliferated method to perform simulations of transport properties on the molecular scale, since the movement of molecules, determined by intermolecular forces, is simulated explicitly. The usage of alternative techniques, such as transition state theory and kinetic Monte Carlo [9], have also been published in the literature. There have been several studies centered around calculating permeation in glassy polymers through simulation [10,11,12,13]. Of relevance to this work are simulations of high free-volume polymers, such as PIM-1 (polymer of intrinsic microporosity) [14,15,16,17,18]. Solubility is usually determined from an adsorption isotherm obtained from a grand canonical Monte Carlo (GCMC) simulation, where the periodic image of the bulk polymer is in virtual contact with a reservoir of the gas of interest. Diffusivity, in turn, is determined from the self-diffusion coefficient of the gas inside the polymer, calculated from the mean square displacement of the gas molecules captured by the trajectories of an MD simulation. The two key properties for gas separation membranes, solubility and diffusivity, are usually not simultaneously calculated, despite equilibrium and transport properties both being obtainable from MD simulations. The solution-diffusion model, which characterizes permeation as a sequential process of adsorption, diffusion and desorption, is used to estimate permeabilities.

There are a number of significant issues with this approach. Firstly, self-diffusion is an approximation for transport diffusion, which is only exact at the zero pressure limit, *i.e.*, at infinitely low gas uptake. Secondly, the solution-diffusion model applies to non-porous polymers, which may exhibit a pore hopping mechanism for diffusing gases, a model incompatible with standard methodologies for calculating diffusivities from molecular simulation.

Frequent ambidirectional collisions of molecules lead to Brownian motion in a bulk fluid. The molecules undergo true random walks, which are the theoretical basis of determining diffusivities from the time derivative of the mean square displacement, also known as the Einstein equation. In this scenario, the mean square displacement (MSD) of the molecules is linearly correlated with time: MSD(*t*) ~ *t* [19]. Such random walks are difficult in a highly porous polymer that features a tortuous network of diffusion paths for penetrant gas molecules. For amorphous polymers, anomalous diffusion, especially at short time scales under 5 ns, is commonly observed [13,20]. Similar to the diffusion inside confined spaces, such as single-file diffusion inside a cylindrical pore, the mean square displacement does not show a linear progression with respect to time; the diffusion is in fact slower. One may express the MSD as a power law, MSD(*t*) ~ *t^γ^*, with the exponent *γ* being equal to one for a linear relationship. For example, in the case of single-file diffusion, the exponent would be 0.5. The diffusion mechanism in microporous polymers is a combination of random unconstrained diffusion and strongly directional confined motion, leading to an exponent between 0.5 and 1. For very long time scales, the diffusion mechanism approaches random Einstein diffusion, but depending on the penetrant gas, this can require simulation times of considerably more than 20 ns. The key challenge for MD simulations is to reach time scales long enough to observe Einstein diffusion and obtain reliable diffusion coefficients from the time dependence of the MSD. Molecular simulation studies of gas diffusion in glassy polymers indicate that it is very challenging to unambiguously distinguish between diffusion mechanisms [21]. It is inherently difficult to determine permeabilities at high accuracy and in a reproducible, transferable manner, both from experiments, as well as simulation approaches. In addition, the independent computational assessment of diffusivity and solubility is a weakness if the determined diffusivities correspond to the zero pressure limit. The combination of these uncertainties leads to large errors [14,15,17] and over-prediction of permeabilities. Alternatively, modeling of small molecule diffusion processes in high free-volume polymers has been performed with techniques based on transition state theory [11,16], but the approach does not yet account for the internal flexibility of the polymer facilitating transport. Recently, an efficient screening of microporous polymer permeability using Monte Carlo solubility simulations in combination with empirical calculations of diffusion coefficients has proven useful for hypothetical PIMs [18].

In an effort to overcome some of these shortcomings, the present study of gas transport in microporous polymers implements a non-equilibrium molecular dynamics (NEMD) methodology in which an external field imposes a pressure gradient between the bulk phases, corresponding to the permeate and retentate. More specifically, the external field is applied at the boundary, and the thermostat is applied only to the solid. The benefit of this refined NEMD approach is that the properties of interest can be directly determined from observables during the simulation, and the critical transport region in the center of the system is free of external forces or thermostats [22]. Similar implementations have been very successful at studying pressure-driven transport in highly complex systems, such as proteins [23], and to study transport in porous ordered structures [24].

In the following manuscript, the methodology to set up the molecular model and its characteristics are outlined, followed by a discussion on developing the non-equilibrium simulation scheme. The simulation results presented thereafter cover the essential gas separation properties, such as permeability and gas uptake, and inquire into the transport mechanism of penetrant gases in PIM-1.

## 2. Molecular Modeling

**Figure 1:**
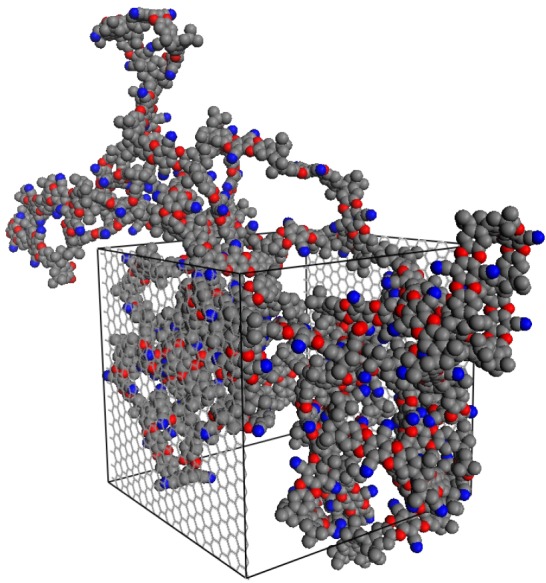
The final polymerized structure of the thin-slice PIM-1 (polymer of intrinsic microporosity) simulation box, PIM-1 (2D). The polymer is polymerized and periodic in the *x* and *y* dimensions, with the *z* dimension being capped by fluid-transparent rigid walls. The polymer is illustrated in unwrapped coordinates; however, periodic boundary conditions were used, with the wrapped coordinates sample shown in Figure 2.

In this study, we construct a polymer slice in contact with bulk gas on either side, which exhibit different pressures during the NEMD simulations due to the external field applied, driving the transport of gas through the polymer. The displacement of the polymer is limited by means of two artificial walls, transparent to the fluid, placed along the *xy* plane (Figure 1). The polymer will thus, on average, retain its bulk properties throughout the simulation. This is a compromise, as an atomistically thin polymer layer subject to gas adsorption would be able to swell, and ultra-thin polymer membranes have been shown to exhibit an enhanced molecule mobility at the free surfaces with significant changes in conformation. In a finite-sized simulation box, modeling this behavior will require excessively large system sizes with no guarantee of accuracy. While one could study a “frozen” polymer configuration, we recognize the need to consider the dynamic rearrangement of the polymer matrix within the confined space dictated by the gas-transparent walls.

### 2.1. Force Fields

The force fields used to simulate the molecular mechanics of the polymer and gas models were taken from available transferable force fields, which have been shown to model the structural and adsorptive properties of PIM-1 with quantitative accuracy [25,26]. The polymer is described by means of a united-atom representation to facilitate computational efficiency with non-bonded interaction parameters taken from the transferable potential for phase equilibria (TraPPE-UA) [27,28,29,30]. To model the flexibility and motion of polymer chains, bonded interaction parameters were taken from the generalized Amber force field (GAFF) [31]. The force field parameters of PIM-1, including atomic partial charges, are listed in [25], and a sample LAMMPS data file containing all bonded and non-bonded interactions for PIM-1 is available for download (see the Supplementary Materials for details). Available models for helium [32] and a flexible model for CO_2_ [33] were used.

### 2.2. Structure Generation

In order to construct an atomistic sample of a slice of an amorphous polymer, the predictive virtual synthesis software, Polymatic [34,35], in conjunction with the LAMMPS simulation package [36], was used. Polymatic has recently been used to simulate a wide variety of polymeric materials, including porous [18,37,38,39] and nonporous glassy [35], linear [40], networked [41,42,43] and composite polymer structures [44]. One of the benefits of using this procedure is the versatility in designing an environment in which the sample is polymerized. To construct the initial simulation sample, a 3D periodic cell of 4.44 nm in edge length is packed with PIM-1 monomers. Rigid walls are placed at the periodic boundaries of the *z* direction of the sample, which resulted in a 3D simulation box of PIM-1 monomers with the restriction that no monomer can penetrate the *z* boundary. To facilitate polymerization, high temperature MD simulations in the canonical ensemble are run at 2,000 K in between bonding steps. During the MD simulations, the walls are held frozen. With the aid of artificial charges (±0.3 *e*) on bonding-capable chain ends, the polymerization proceeds until no more bonds can be made within a reasonable time frame. Here, we employ 250 cycles of 5 ps MD simulations. The result is a polymerized PIM-1 sample that resembles a 4.5-nm thin slice of polymer, as the polymer is not periodic in the *z* dimension. The final simulation configuration is shown in Figure 1.

**Figure 2:**
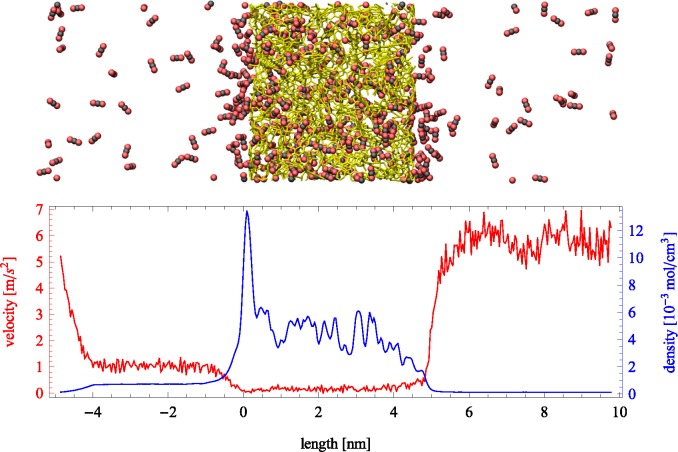
(Top) Snapshot of an instantaneous configuration of the simulation cell. Periodic boundary conditions are applied in all directions and an external force applied to the fluid molecules in the left boundary. Carbon dioxide molecules are depicted by black and red, while a stick representation of a slab of PIM-1 is shown in yellow. (Bottom) Average velocity profile in the direction of transport (red) and the fluid density profile (blue) across the simulation cell. Density within the porous region is referred to the total volume, including the polymer.

### 2.3. Model Characterization

The physical properties of the polymer sample were characterized by means of bulk and skeletal density, fractional free volume, geometric surface area and pore size distribution. To calculate these properties, the rigid walls were removed, and the box dimensions were corrected to account only for space occupied by the polymer (*a* = *b* = 4.44 nm, *c* = 4.53 nm). The simulation density (*ρ*_bulk_) was determined as the ratio of the total mass *m* to the total volume of the simulation cell *v*_total_, including all of the pore volume (*i.e.*, *L*^3^, where *L* is the box length). However, the skeletal density (*ρ*_skel_) corresponds to the skeletal volume of the polymer matrix *v*_skel_ or the total volume minus the pore volume (*i.e.*, *v* = *L*^3^ − *V*_pore_). In these simulations, the pore volume is calculated as the amount of volume accessible to a helium-sized probe by means of a Widom insertion method, which is consistent with experimental helium porosimetry. The bulk density and the skeletal density are related via:

(1) $$\frac{1}{\rho_{\text{skel}}}=\frac{1}{\rho_{\text{bulk}}}-\frac{v_{\text{pore}}}{m}$$

The fractional free volume (*f*) was calculated as *f* = 1 *−* 1.3(*V*_vdW_*/V*_sp_), where *V*_vdW_ is the volume occupied by the united atom beads of the polymer in the simulation sample, and *V*_sp_ is the specific volume calculated as the reciprocal of the density (1*/ρ*_bulk_). *V*_vdW_ was calculated by subtracting the simulation void volume (*φ*) from *V*_sp_. *V*_vdW_ is defined as the point where the intermolecular potential is null for each bead in the simulated sample. Consequently, the simulation void volume was calculated by employing a probe molecule of 0.0 nm and accounting for the regions of space where the probe experiences a repulsive interaction. Similarly, the geometric surface area (SA_geo_) was calculated as the amount of surface area outlined by the center of a N_2_-sized spherical probe molecule, *d*_N2_ = 3.681 Å. The pore size distribution (PSD) is the numerical derivative of the cumulative pore volume function *V* (*r*) with respect to probe size, *r*. All porosity characterizations were calculated using the Pore Blazer code [45].

Molecular simulations of PIM-1, using the same force field model used here, have been shown to resemble the physical polymer in properties, such as BET surface area, adsorption isotherms, enthalpies of adsorption, gas selectivities and wide angle X-ray scattering experimental data, in previous works of the authors [18,26,35,37,46]. Moreover, the results of the structural characterization of the PIM-1 (2D) model is in excellent agreement with the average values of the previous 3D periodic PIM-1 simulated samples (“PIM-1 (sims.)” [26]) and available experimental data (“PIM-1 (exps.)”) for a range of porosity characteristics, as shown in Table 1. The bulk and skeletal density of PIM-1 (2D), 0.916 and 1.38 g/cm^3^, were in excellent agreement with the values reported for the previous PIM-1 (sims.), 0.93 and 1.32 g/cm^3^, respectively. When comparing these values to reported experimental results, the distinction between bulk and skeletal density is important for porous materials. Experimental investigations of PIMs typically report skeletal density, which depends not only on the density of the sample, but also on the probe used to calculate the pore volume. Both of these factors contribute to the large spread of experimentally observed values, which span from ~1.05–1.4 g/cm^3^. However, both the PIM-1 (2D) and previous PIM-1 (sims.) skeletal density values fall within this range. It is worth noting, therefore, that density alone is not a sufficient means to validate the simulated sample of high free-volume polymers. Thus, additional porosity characteristics were evaluated, namely the fractional free volume, surface areas, void volume and pore size distributions.

**Table 1:** Porosity characteristics of PIM-1 simulated (sims.) and experimental (exps.) samples.

Sample	*ρ*_bulk_	*ρ*_skel_	*f*	SA_geo_	SA_BET_	*φ*	Reference
			
	(g*·*cm^−3^)		%	(m^2^*·*g^−1^)		(cm^3^*·*g^−1^)	
PIM-1 (2D)	0.916	1.38	25.8	530	–	0.469	this work
PIM-1 (sims.)	0.93 (0.02)	1.32	24.3 (1.3)	595 (85)	830	0.448 (0.019)	[26]
PIM-1 (exps.)	–	1.056–1.4	24–26	–	760–875	–	[47,48,49,50,51]

The standard deviation of the molecular simulations of PIM-1 from [26] is reported in parentheses for the PIM-1 (sims.) sample, and PIM-1 (exps.) reports the range of available experimental data.

The amount of fractional free volume of the PIM-1 (2D) sample was calculated to be 25.8%, which compares well with the bulk PIM-1 simulation value of 24.3% ± 1.3%, both of which fall directly within the range of experimentally reported values, 24%–26%. In addition, the geometric surface areas of the simulated samples agree well, with PIM-1 (2D) and the previous PIM-1 (sims.) exhibiting 530 and 595 m^2^/g of surface accessible to a spherical N_2_-sized probe. As was previously shown in [26], the geometric and BET surface areas do not agree for PIM-1 as a result of several assumptions within BET theory; however, as the simulated BET value of the previous PIM-1 (sims.), 830 m^2^/g, compares well with the range of experimental BET surface area, 760–875 m^2^/g, the PIM-1 (2D) sample of this study should compare equally, as well. Lastly, the void volume reported in Table 1, which is the amount of physical space of the simulated model unoccupied by the polymer, is similar between the previous PIM-1 (sims.) and the PIM-1 (2D) model at 0.448 and 0.469 cm^3^/g, respectively. As a result, the density of the PIM-1 (2D) model, the fractional free volume, the surface areas and the void volume all compare well with the previous PIM-1 (sims.) model and available experimental data.

**Figure 3:**
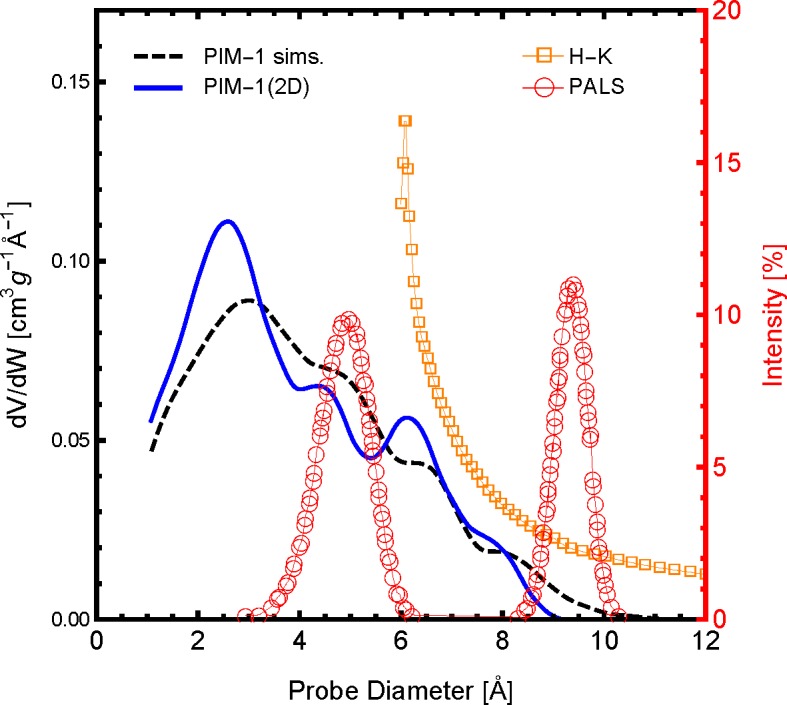
The pore size distribution of the two-dimensional PIM-1 sample used here (PIM-1 (2D)), in a solid blue line, compared with the molecular simulations of [26] (dashed black line). Also shown are available pore size distributions of experimental PIM-1 samples using positron annihilation lifetime spectroscopy (PALS) of PIM-1 in the powder form in red open circles [52] and using the Horvath–Kawazoe (H-K) method applied to N_2_ adsorption at 77 K; H-K in orange open squares [50]. The PALS data are reported in percent intensity according to the right axis.

The distribution of pore sizes within the framework is arguably the most influential polymer sample characteristic of gas permeation. The pore size distribution (PSD) profile of the polymer slice model, PIM-1 (2D), and 3D simulation samples compared to available experimental data are shown in Figure 3. As one can see, both the simulations and experiments show that a significant amount of the pore size distribution exists below 10 Å, although with slightly different distribution profiles. For the PIM-1 (2D) and the previous PIM-1 (sims.), the frequency and distribution of pore sizes are in excellent agreement. When comparing between simulated model PSDs with experimental PSDs, one must be aware of the method and interpretational models used to generate the experimental data. Shown in Figure 3 are two available experimental PSDs of PIM-1, which were generated using the Horvath-Kawazoe (H-K) method [50] and positron annihilation lifetime spectroscopy (PALS) [52]. The H-K method uses a slit pore model applied to the N_2_ adsorption isotherm at 77 K, and the distribution profile agrees remarkably well with the PIM-1 simulation models. Although, the H-K method of PSDs is limited by both the size of a N_2_ molecule and the low pressure limit of the equipment, the smaller pore sizes shown in the simulations were not observed. The existence of pores below 6 Å is confirmed by the PALS experiment. However, these data were interpreted with a bimodal distribution, which was not observed in either the simulation models or the H-K PSDs. Although the comparison between these different means of generating a PSD is not perfect, it may be concluded that the PIM-1 (2D) model has a reasonable PSD compared to both previous simulations and experimental data. As such, the PIM-1 (2D) model should exhibit similar gas permeability characteristics as a similarly-sized element of a bulk PIM-1 membrane as a result of the these similar pore structure characteristics.

### 2.4. NEMD Simulations

**Figure 4:**
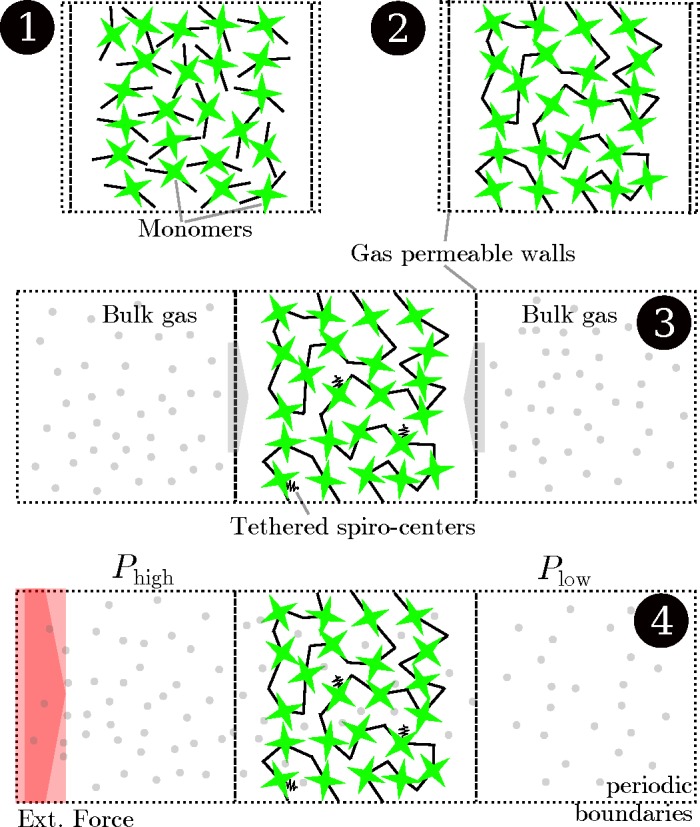
Schematic drawing of the simulation process. Initially, a simulation cell with two walls confining one specific dimension is populated with polybenzodioxane monomers (1). The sample is polymerized to create the PIM-1 2D sample (2). The box is elongated in the dimension of the two walls, which are made permeable for gases. While some selected carbon atoms on the polymer backbone are tethered to keep the structure from shifting inside the wall, the gases pass through the walls and adsorb to the polymer from the bulk regions during an equilibration run (3). Finally, an external field is imposed on the boundary, creating regions of higher and lower pressure on either side of the polymer. The steady-state flux and pressure difference are measured during this non-equilibrium molecular dynamics (NEMD) run (4).

The walls confining the polymer in the *z* direction are made permeable to gas molecules, and two regions filled with gas molecules are constructed next to the polymer sample, creating an elongated simulation box in the *z* direction three-times the size of the original polymer sample (see Figure 4). Preliminary MD equilibration runs were performed to allow the gases to adsorb to the polymer. As gases saturate the polymer during equilibration, the gas uptake, which depends on the bulk pressure, was measured directly by integrating the gas density distribution:

(2) $$c(P)=\frac{1}{V_{P}}\int \rho (z)dz$$

where *ρ* denotes the gas density and *V*_P_ denotes the volume of the polymer, which will specifically depend on the integration boundaries multiplied by the height and depth of the simulation box (in *x* and *y* directions, respectively). As expected, inert helium exhibits modest adsorption, while carbon dioxide adsorbed strongly. The gas regions initially contained 56 and 330 molecules for helium and carbon dioxide, respectively, such that the bulk gas pressure at equilibrium was in the order of 10 bar.

An external field applied to a thin slab (0.3 nm in thickness) at the boundary of the simulation box perturbs the system from its equilibrium state. The field acts like an acceleration in the *z* direction on the gas particles at the boundary and creates regions of higher and lower bulk density on opposite sides of the polymer slice. Periodic boundary conditions are applied in all three dimensions, so the two bulk regions are in direct contact, but kept at different pressures, due to the external perturbation. The difference in pressure drives molecules to permeate through the polymer matrix, so that a steady net flux of gas molecules develops. During this steady-state run, the net gas flux and pressure difference between the two bulk regions are measured. Permeability, *K*_P_, can then be determined by invoking Darcy’s law:

(3) $$J=K_{P}\frac{\Delta P}{\Delta z}$$

The fixed walls keep the polymer in place in the direction of transport (*z* direction). In order to prevent perpendicular drifting of the polymer within the confining sheets, six carbon atoms of the entire polymeric sample (from central spirobisindane groups) were tethered to their initial positions with a spring constant of *k_s_* = 5 kcal/(mol Å^2^). During the full length of the simulation, the polymer is allowed to move, subject to the tethering of these six sites, *i.e.*, we are not modeling a static structure, but rather, a flexible one. Notwithstanding, due to the inherent rigidity of the PIM, the initial conformation is not seen to change significantly.

The external field performs work on the gas molecules, which must dissipate as heat to maintain isothermal conditions, and hence, needs to be removed from the system in order to maintain a steady-state response. To this end, the Nosé–Hoover thermostat at 298 K was used for the polymer, while the gas was left without a thermostat and left to release the additional energy through interactions with the polymer. This protocol is followed in order to prevent unphysical perturbations arising from thermostatting from being introduced during the transport process. It is important here to balance the magnitude of the external field with the system’s ability to release heat through gas-polymer collisions. A set of field strengths, ranging from 0.01179 to 0.0472 kcal/(mol Å), are applied. For helium, the field was also applied in the opposite direction, that is between *−*0.01179 and *−*0.0472. For CO_2_, the field was applied to only the carbon center at these magnitudes during one set of simulations, and during another set of simulations, it was applied to all three sites of the CO_2_ molecule. This results in the magnitude of the external field being three-times as strong.

The system is inhomogeneous, so the NEMD simulations were run without Ewald summations to account for long-range electrostatic interactions. Instead, Coulombic interactions are cut off at 15 Å. This implies that the pressure of the gases in the bulk is lower compared to gases of the same density with full account of the long-range Coulombic interactions.

Simulations were started by allowing the gases to saturate the polymer and reach their equilibrium state in the bulk, *i.e.*, no external field is imposed at the boundary. The bulk gas pressure at equilibrium resulted in 10.19 bar for helium and 9.12 bar for carbon dioxide. Equilibrium solubilities were determined from the gas uptake at the respective pressure, *S*(*P*) = *c*(*P*)*/P*. For helium, 0.065 mmol/cm^3^ were adsorbed by the polymer, and for carbon dioxide, the uptake resulted in 4.73 mmol/cm^3^. This corresponds to solubilities of 0.14 and 11.77 cm^3^ (STP)/cm^3^ bar, for helium and CO_2_, respectively. These results are in good agreement with gas solubilities determined experimentally [51,53,54], which are reported below 0.25 cm^3^ (STP)/cm^3^ bar for helium and between 11.2 to 11.3 cm^3^ (STP)/cm^3^ bar for CO_2_. After these initial MD equilibrations of 3 ns, which allowed the polymer to saturate with gas, NEMD simulations were performed for 2 ns for the system to reach steady state and subsequently 10 ns to gather statistics. The time step was set to 1 fs.

## 3. Results and Discussion

NEMD simulations are reported for pure helium and carbon dioxide. Helium, being a small, light and relatively inert gas at the conditions studied here, has a high diffusion coefficient in PIM-1 with little adsorption, while carbon dioxide strongly adsorbs to the polymer and exhibits low diffusivity. The steady-state response of the system during a typical NEMD simulation can be observed in Figure 2, where the region on the left exhibits a higher density than the one on the right. Velocity profiles present the opposite behavior due to mass continuity. The spikes in density shown in the lower part of Figure 2 (blue line) showcase the strong adsorption of carbon dioxide on the polymer. The profile is shown for the largest force applied (*f*_ex_ = 0.0472 kcal/mol Å). Directly observable from the plot are the differences in density (which relate to pressure differences) between the two bulk sections, as well as the average velocity. The effective flux can be determined from the averages of density and velocity as follows:

(4) $$J_{z}={\bar{v}}_{z}\bar{\rho }$$

Pressure differences can be obtained from the NEMD simulations directly via the virial route [55] or by calculating a bulk isotherm and determining the pressure from the measured densities, which was the preferred option in this study.

**Figure 5:**
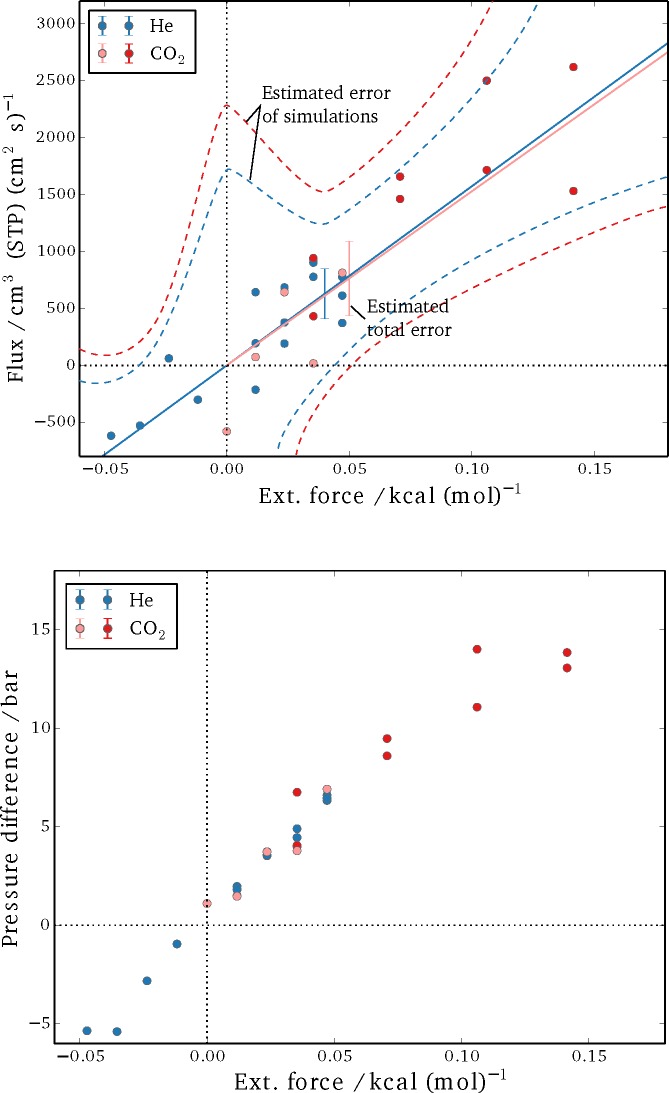
Simulation results for steady-state flux (Top) and the pressure difference between bulk sections (Bottom). Blue circles show the results for helium; red circles show the results for carbon dioxide (dark red represents simulations in which all three sites of the CO_2_ molecules were perturbed, and light red symbols show simulations in which only the carbon center felt the external force). The dotted lines in the plot on the left are estimations for the error expected in a single simulation, while the error bars represent the total error of the estimated steady-state flux from simulations at different forces.

The results for the steady-state fluxes and pressure differences of the performed sets of simulations are shown in Figure 5. It can be seen that the spread is considerable for the detected net flux, in particular. A linear fit for flux as a function of applied external field was obtained from the data and is plotted as a solid line for both helium and CO_2_. Subsequently, the absolute difference between the individual results and the fit are summed up and averaged over the number of simulations, yielding an estimate for the total error (*δ* = 1*/N*_sim_ ∑*|J*_sim_ *− J*_fit_*|*, with *N*_sim_ being the total number of simulations and *J* denoting the respective fluxes). The dashed curves in Figure 5 are a qualitative estimate of the expected error for individual runs, but they were not specifically quantified. The reasoning is that the non-equilibrium effects add up as a higher external force is imposed, and a larger deviation from the linear-response regime is to be expected. Meanwhile, it is difficult to obtain a single reliable measurement for very small external forces, as the net fluxes are very small; thus, the signal-to-noise ratio is low, and extremely long simulations would be required. By varying the external field, a balance between these two extremes is achieved.

**Figure 6:**
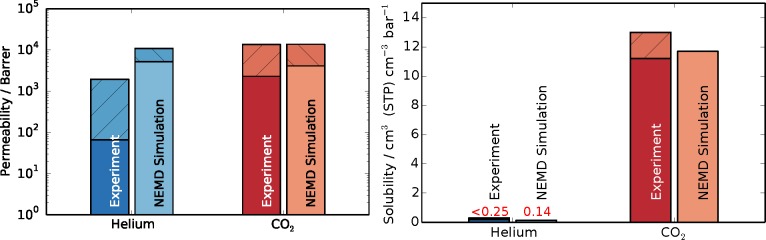
Permeability and solubility results from NEMD simulations compared to experimental results for helium in blue and carbon dioxide in red [51,53,54,56,57]. The shaded area of the bar chart shows the spread of experimental data and the estimated error for simulations results in the case of permeability. Solubilities are obtained from a single initial equilibration, and since their calculation is very robust, errors for solubility were not estimated.

Upon obtaining the steady-state flux and pressure difference, Equation (3) was employed to determine the permeabilities, with Δ*z* being equal to the thickness of the polymer slice (4.53 nm). The results in comparison to experimental data are shown in Figure 6. The shaded areas on top of the bars denote the spread in reported values from experiments and the estimated uncertainty of the simulations. Since solubilities were obtained from the initial equilibration of the simulations, their results are not accompanied by error estimations. Experimental data were reported from time-lag and gas chromatography experiments of PIM-1 membranes [51,53,54,56,57]. The spread in experimental results for permeation presumably stems from the different methods and solvents used in casting of the membrane and subsequent treatments. The permeability of carbon dioxide obtained in this work, through the thin slice model polymer, is in very good agreement with experimental data, lying at the upper end of the data range. The result obtained for helium in this work is above the spread of experimental data. The cause of this over-prediction might be found in the fact that hydrogen atoms are not explicitly modeled, but lumped together as a group with their bonded carbon atoms, resulting in a smoother structure, as opposed to the full-atomistic detailed model. In other words, a smoother force field exhibits higher diffusivity, because there is less molecular friction within the system. Additionally, the interfacial regions could also contribute to higher transport by exhibiting more free volume than the bulk regions.

**Figure 7:**
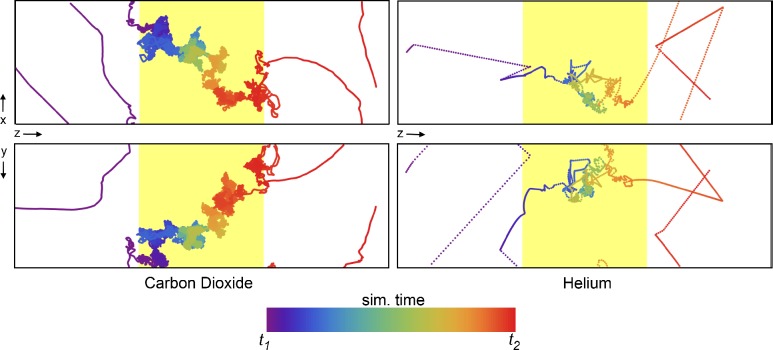
The movement of a single CO_2_ molecule (left) and He atom (right) through the polymer matrix (yellow shaded region). Each point represents the location of a molecule at every 0.5 ps of simulation time, with the color of the points scaled according to the relative time (see color bar). The simulation time was selected such that only a single permeation event of the many occurring during the 10-ns simulation is shown. Δ*t* = *t*_1_ *− t*_2_ is 3.4 ns for CO_2_ and 80 ps for Helium. The top and bottom plots represent the XZ and YZ projections, respectively.

The NEMD approach closely mimics how experiments are conducted to characterize membrane separation performance, yielding a macroscopic view of the gas permeation through the polymer. However, it also allows further investigation of effects influencing transport on the molecular scale with an abundance of detailed information not available through physical experiments. The specific diffusion paths for each gas molecule individually can be obtained from the simulation trajectories. One such trajectory is plotted in Figure 7, where the time dimension is color coded. It starts at purple and blue and progresses through the color spectrum to yellow and red. The trajectory shown is a small excerpt from the full 10-ns trajectory to showcase a single permeation event through the polymer slice. The differences between the permeation of carbon dioxide and helium become apparent in Figure 7. The diffusion of carbon dioxide is slowed down by frequent and complex interactions inside the polymer, and in the event shown in Figure 7, the passage takes 3,400 ps compared with only 80 ps for helium. As expected, a helium atom interacts very little with the polymer and finds a path through the polymer matrix much more quickly than a CO_2_ molecule. Both pathways suggest that molecules within the polymer matrix spend a considerable amount of time in “random walks” throughout the extent of the available free volume, *i.e.*, the mechanism deviates from a simple “pore hopping” trajectory expected for a dense polymer as a result of exhibiting highly interconnected porosity. The occurrence of pore hopping is likely proportional to the amount of pore volume, and thus, high free-volume polymers exhibit fewer pore hopping events during permeation. Penetrant molecules that plasticize the polymer matrix to a greater extent may induce further deviation from a hopping mechanism. It has been suggested that alcohols and alkanes, which considerably swell the PIM-1 membrane, are well described by a convective transport model and exhibit pore flow transport [58]. The extent to which pore flow contributes to the permeation of these gases in PIM-1 has, however, yet to be quantified.

Experimentally determined diffusion coefficients are in the order of 100 and 3000 ×10^−8^cm^2^/s for CO_2_ and helium, respectively, although reported diffusivities can vary as much as the permeabilities do. If we define a characteristic length in order to compare diffusion processes as $L=2\sqrt{D_{\tau }}$, in analogy to a steady diffusion process into a semi-infinite space, where *D* is the diffusion coefficient and *τ* the time span, we can determine this diffusion length based on the duration of the passages in Figure 7. With 3.4 ns for CO_2_ and 80 ps for helium, this results in diffusion lengths of 3.7 nm for CO_2_ and 3.1 nm for helium, numbers that compare well with the thickness of the polymer at 4.5 nm.

**Figure 8:**
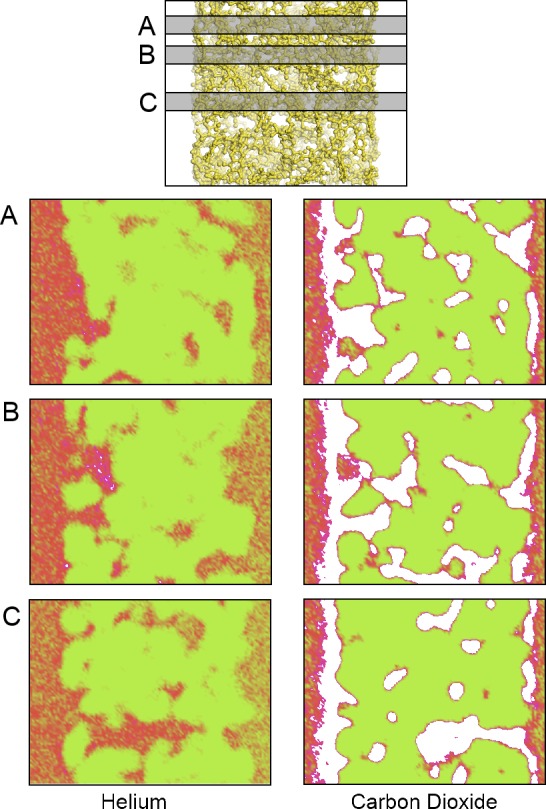
A density map showing the density of locations for the (left) He atoms and (right) CO_2_ molecules on an XZ projection calculated at three different Y slices: (A) 0.675; (**B**) 1.275 and (**C**) 2.775 nm; from top to bottom, respectively. The plots show areas of high (white), average (red) and low (green) gas density. The top model shows the relative location of the polymer and the corresponding plane locations. Only a small fraction of the bulk fluid sections is depicted to the left and right of each plot. An animated version of this visualization can be found in the Supplementary Material.

An aggregate perspective on the diffusion paths underlines this aspect further. Figure 8 shows three slices of the density distributions accumulated over an entire 10-ns simulation in the *xz* plane. The regions occupied by the polymer that are not accessible to the gas molecules are depicted in green. They are interfused by red, denoting an average occupation, and white areas, which denote frequently explored diffusion channels for the gases. These channels are highly connected and build a percolated diffusion path through the polymer. This path is very tortuous, but it percolates from one bulk region to the next. The density plots (Figure 2) and density distribution maps (Figure 8) show an enhanced excess adsorption of CO_2_ at the solid-fluid interface. This behavior is commensurate with the interfacial properties of CO_2_ at room temperature and the high pressures employed.

The influence of free volume on the permselectivity and permeability of gases in glassy polymers is the focus of a number of recent studies with the objective of pushing the trade-offs between selectivity and permeability past their current thresholds.

When measuring permeability experimentally, one has the option to measure either time-lag diffusion or secant solubility and invoking the solution-diffusion model (*S* = *P/D* or *D* = *P/S*) to obtain the other property of interest. Robeson *et al.* [59] noted a discrepancy between these two alternative routes and showed that the deviation between diffusion coefficients in PIM-1 depends on pressure and can amount to more than 50% in the pressure range 8–12 bar. Similar trends were observed by Li *et al.* [54] upon measuring the temperature and pressure dependence of permeability in PIM-1. They found a decline in carbon dioxide permeability in the pressure range from 1–10 bar of up to 25%. These observations underline the inherent difficulties associated with the determination of the permeability, diffusivity and solubility of gases in high free-volume polymers, both experimentally and through simulation. A crucial assumption is that the sorption process and several transport phenomena are all sequentially related, yet independent. NEMD simulations follow the experimental approach very closely and, in analogy, are capable of obtaining permeabilities and solubilities.

Li *et al.* point out that as “the molecular picture of the solid-state structure is still emerging” [54], the main reason for high permeability through PIM-1 and other glassy high free-volume fractions is the high diffusion coefficients. The simulation data concur with this viewpoint by highlighting the considerable diffusion path accessibility inside the polymer. While dense glassy polymers are situated at one end of the spectrum, exhibiting a common pore hopping diffusion mechanism and structured materials with sieving and Knudsen-type separation mechanisms at the other end of the spectrum, the separation mechanism in high free-volume polymers seems to simultaneously exploit the tortuosity of its diffusion path and the energetic interaction between the polymer solid and permeating gases. Mixed-matrix membranes of glassy polymers and structured molecular materials, such as metal-organic frameworks, are garnering interest for improved separation performance by tapping into both regimes [60]. The method herein presented would be ideally suited to study in detail the transport dynamics of such inhomogeneous materials. It is well known that physical aging, the residues of casting solvents, humidity, plasticization and swelling can influence permeation significantly. *In silico* experiments are ideally clean, and any of these mentioned effects can or should be controlled individually. NEMD permeation experiments could be further employed to investigate the details of how the presence of pollutants can influence transport dynamics at the smallest scale.

## 4. Conclusions

This study presents the first explicit modeling of permeation through a high free-volume polymer. As opposed to measuring self-diffusion coefficients and invoking the solution-diffusion model to obtain permeation properties, this study obtains a picture from an atomistically detailed simulation of direct gas permeation through a slice of PIM-1 polymer. The thin polymer slice was generated by the Polymatic algorithm and compares very well with 3D-periodic simulated samples in terms of porosity and pore size distributions. By confining the generated structure between rigid walls, the polymer slice is forced to keep its structure, resembling the 3D periodic image of a bulk. With gas regions placed on either side of the slice, direct permeation simulations were performed by applying a non-equilibrium scheme. In analogy to experimental measurements, permeabilities were calculated from the steady-state flux and pressure gradient. As the simulation is initiated with an equilibrium simulation to allow the gases to saturate the polymer before applying external perturbations, adsorption characteristics can also be calculated. Although there are large uncertainties in the experimental results for gas permeability in PIM-1 and the molecular interaction force fields are not fitted explicitly to transport properties, the quality of agreement is good. Furthermore, the information obtained from NEMD simulations sheds light on the diffusion mechanism. In this case, a deviation from a straightforward gate-hopping mechanism was observed. Moreover, the present approach allows for a range of crucial phenomena to be investigated *in silico*. Most notably, the approach lends itself to the simulation of mixtures and complex organic molecular fluids, as well as composite and inhomogeneous membranes. Similarly, the approach could be refined to account for swelling of the polymer [61].

## Supplementary Information

Provided in the electronic supplemental information are:

- The LAMMPS script file, defining the parameters and procedure of the simulation;
- Visualization of the gas density distribution maps through the polymer for *yz* planes of the entire polymer, in effect an animation of Figure 8.

Furthermore, a LAMMPS data file of PIM-1 is available from the force field database at: [62]
